# Comparative Study of Conduction Mechanisms in Disodium Phthalocyanine-Based Organic Diodes for Flexible Electronics

**DOI:** 10.3390/molecules25163687

**Published:** 2020-08-13

**Authors:** Leon Hamui, María Elena Sánchez-Vergara, N. Díaz-Ortega, Roberto Salcedo

**Affiliations:** 1 Engineering Department, Universidad Anáhuac México, Avenida Universidad Anáhuac 46, Col. Lomas Anáhuac, Huixquilucan, Estado de México 52786, Mexico; leon.hamui@anahuac.mx (L.H.); nelia.diazortega@hotmail.com (N.D.-O.); 2 Instituto de Investigaciones en Materiales, Universidad Nacional Autónoma de México, Circuito Exterior s/n, Ciudad Universitaria, Coyoacán 04510, Mexico; salcevitch@gmail.com

**Keywords:** thin film, DFT calculations, electrical properties, bandgap

## Abstract

In the current work, flexible diodes with flat heterojunction and dispersed heterojunction architecture were manufactured with to study the behavior of thin films of disodium phthalocyanine (Na_2_Pc). The thin film devices, using the electronic acceptor tetracyano-π-quinodimethane (TCNQ), were fabricated by high-vacuum thermal evaporation with annealing post-treatment in order to optimize their behavior. Theoretical calculations based on density functional theory (DFT) with dispersion force analysis were carried out in order to simulate molecular interactions and to establish the nature of the weak interactions between the Na_2_Pc and TCNQ fragments. In the optimized structure of the coupled Na_2_Pc-TCNQ, the electronic relationship between phthalocyanine and TCNQ was observed to be through hydrogen bonds with bond lengths of 2.94 and 3.13 Å. Dispersed heterojunction device current density values were considerably larger than those of the flat heterojunction device. Barrier heights of 1.024 and 0.909 eV and charge mobilities of 10^−10^ and 10^−9^ m^2^/Vs for the flat heterojunction device and the dispersed heterojunction device, respectively, were observed. A small effect was observed on the electrical properties by thermal annealing on the flat heterojunction device. The threshold voltage decreased from 1.203 to 1.147 V and φ_b_ decreased by 0.001 eV.

## 1. Introduction

The design of electronic devices has motivated the search for organic semiconductors of rapid processing which have high-density data stocking [1], photovoltaic applications [2], and low cost. Organic semiconductors arise from molecules that are highly conjugated and display rich π electrons. One of the families of these kind of compounds is the metal phthalocyanines (MPcs), which are important because their π orbitals are energetically accessible for charge transport. Once a charge enters an MPc molecule, rapid delocalization occurs throughout the macrocycle. This gives rise to a rapid conduction inside the MPc, but even more importantly, thanks to the molecule’s planarity, charge delocalization eases among several MPc molecules due to spatial overlapping with the adjacent molecules’ electronic states. MPcs are compounds known since 1907 and are used mainly as dyes [1]. The development of MPcs and their unique structural, electrical, and optical properties was important due to the discovery of their semiconductor functionality in recent years. These characteristics resulted in their utilization in various electronic devices applications, such as organic field effect transistors, organic light emitting diodes, gas sensors, supercapacitors, spintronics, electrocatalysis, magnetic resonance imaging, and others [2,3,4,5,6,7,8,9,10]. MPc semiconductors have high hole mobilities (μ) [11], for instance, the CuPc μ is between 10^−8^–10^−7^ m^2^/Vs [12,13,14] and is high enough for employment as a p-semiconductor on photovoltaic devices [11,12,13,14,15], whereas the ZnPc μ value is around 10^−10^ m^2^/Vs [16]. Copper and zinc MPcs are the most commonly used MPcs in electronic devices; in films, the value of μ perpendicular to the device substrate was found to be 10^−9^–10^−8^ m^2^/Vs [11,13,17]. For electronic device applications, MPcs should be prepared as thin layers with easily reproducible structures and ordering [18]. Interest in their polycrystalline film structures is primarily due to their comparatively high charge-carrier mobility [18], intense red and near IR absorbance, and high thermal and photo stabilities [19,20]. Additionally, MPc films may be deposited from either their vapor phase or solution [21]; it is relatively easy to deposit MPcs films, which adhere well to substrates. As a material requirement, for the most basic architecture of an MPc devices, a transparent substrate is included. The most frequently used substrate for a variety of reasons is glass, because it provides an oxygen-water diffusion barrier for the MPc device. However, the use of flexible substrates, like poly(ethylene terephthalate) (PET) have advantages associated with polymeric materials, such as flexibility, high mechanical properties, low-temperature large-area processing, and roll-to-roll deposition techniques [11]. For vapor-deposited MPc devices, deposition on glass or plastic substrates is equally feasible; however, few studies have been conducted on plastic substrates thus far.

MPc molecules in films may attain face-on or edge-on orientation with respect to the substrate plane while forming monoclinic and triclinic forms [22]. The monoclinic form presents strong absorption in the visible range while the triclinic form exhibits intense IR absorptions bands [22,23]. These crystalline shapes and their influence on the film’s electrical behavior were widely studied in M(II)Pcs. Most M(II)Pcs have a planar structure and exhibit polymorphism, including the monoclinic and triclinic forms [24]. The structural and electrical behaviors of some M(II)Pcs like PbPc were studied, revealing that they contain large metallic ions that generate distortion in the molecule. This is because the size of the ion exceeds the size of the macrocycle cavity, therefore the Pb ion is outside the plane of molecule and the PbPc adopts a “shuttlecock” shape [24]. However, M_2_Pc molecules like Na_2_Pc have not been studied as extensively. Sodium ions are above and below the plane of the molecule, thus the phthalocyanine loses planarity but gains conductivity. Because of this, in the present work, Na_2_Pc thin films were studied as part of the electronic devices deposited on PET. The devices were manufactured with flat heterojunction and dispersed heterojunction architectures using the electronic acceptor tetracyano-π-quinodimethane (TCNQ), which was previously used by some authors of this work [25]. The aim was to generate charge transport between the Na_2_Pc molecule, or hole carrier, and the TCNQ within both devices. TCNQ has interesting electrical properties due to its low dimensionality and energy-band formation, as well as its ability to yield or accept electrons at a low energy cost, resulting in a partially occupied higher energy band [25]. To optimize the electrical behavior of phthalocyanine films, an annealing treatment was conducted and the optical band gap was evaluated before and after the treatment. In addition, DFT (density functional theory) calculations were carried out in order to search for an Na_2_Pc-TCNQ pathway interaction to obtain highest energy occupied molecular orbital–lowest energy unoccupied molecular orbital (HOMO-LUMO) orbital energy values and the theoretical band gap, which was compared with the experimental one. The latter gives evidence of useful information regarding the analysis of Na_2_Pc behavior in an electronic device.

## 2. Materials and Methods

**Figure 1:**
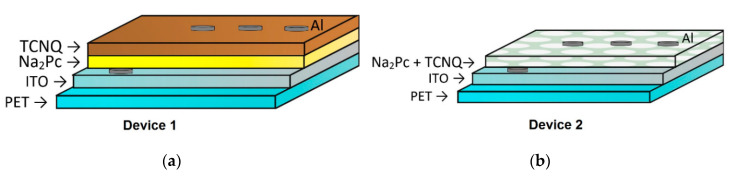
Structure of the (**a**) flat heterojunction and (**b**) dispersed heterojunction devices.

The Na_2_Pc thin film devices were fabricated using a high-vacuum thermal evaporation system (HVTE) onto different substrates. The used evaporation source was a tantalum boat and the temperature was slowly increased temperature to 498 K via a ramp. The selected pressure in the vacuum chamber was 1 × 10^−5^ torr before film deposition. The evaporation rate depended on the semiconductor melting temperature and was of 0.4 Å/s, thus requiring a film thickness of 77.6 nm. The latter was obtained by a quartz-crystal microbalance monitor connected to a thickness sensor. The Na_2_Pc (disodium phthalocyanine: C_32_H_16_N_8_Na_2_) and the TCNQ (7,7,8,8-tetracyanoquinodimethane: C_12_H_4_N_4_) were obtained from Sigma-Aldrich (Saint Louis, MO, USA) and required no further purification. Commercial indium tin oxide (ITO) (In_2_O_3_·(SnO_2_)_x_) coated polyethylene terephthalate film (PET-ITO) (100 Ω/sq), quartz, and monocrystalline silicon substrates were used for sample deposition and further characterization. Before deposition, all substrates except for PET-ITO were cleaned under an ultrasonic process using organic solvents (chloroform, methanol, and acetone) and followed by drying in a vacuum. Two devices were manufactured using different processes (Figure 1): (i) Na_2_Pc and TCNQ were used in separate source ports for consecutive deposition (PET/ITO/Na_2_Pc/TCNQ/Al), and (ii) a mixture of Na_2_Pc-TCNQ was codeposited with a 1:1 stoichiometry obtaining (PET/ITO/Na_2_Pc+TCNQ/Al). Additionally, thermal annealing at 90 °C for 1.5 h was conducted on the electronic devices to crystallize the films. Infrared (IR) spectroscopy analysis was carried out on a silicon substrate sample, which helped to verify the formation of chemical bonds and functional groups with a Nicolet iS5-FT spectrometer (Thermo Fisher Scientific Inc., Waltham, MA, USA) on a wavelength range of 4000 to 500 cm^−1^. UV-Vis spectrometry allowed for measurement of the optical transmittance and absorption in the 1100–200 nm range using a UV-Vis spectrophotometer (Thermo Fisher Scientific Inc., Waltham, MA, USA) on a quartz substrate sample. The electrical properties were obtained using a sensing station with a lighting controller circuit from Next Robotix (Comercializadora K Mox, S.A. de C.V., Mexico City, Mexico) and an autoranging Keithley 4200-SCS-PK1 pico-ammeter (Tektronix Inc., Beaverton, OR, USA).

## 3. Computational Details

The optimized structure of Na_2_Pc was obtained by applying the density functional theory (DFT) method. The interaction between Na_2_Pc and TCNQ was also calculated. From these calculations, the energy values of the highest energy occupied molecular orbital–lowest energy unoccupied molecular orbital (HOMO-LUMO) set and the bandgap were obtained. All calculations were carried out by applying the DFT method based on the combination of Becke’s gradient corrections [26] for exchange and Perdew-Wang’s for correlation [27]. This is the scheme for the B3PW91 method, which forms part of the Gaussian16 [28] Package. The calculations were performed using the 6–31G** basis set. Frequency calculations were carried out at the same level of theory to confirm that the optimized structures were at the potential surface minimum. The hydrogen bonds were studied by means of atoms in molecules (AIM) theory [29] by using the AIMPAC package [30], and the dispersion Grimme correction (G-3) was carried out using the DFT-D3 method [31].

## 4. Results and Discussion

### 4.1. DFT Study

**Figure 2:**
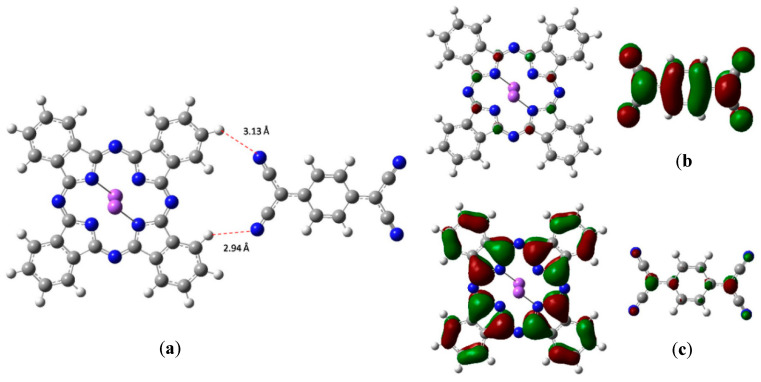
Optimized structures of Na_2_Pc+tetracyano-π-quinodimethane (TCNQ): (**a**) Hydrogen bonds, (**b**) lowest energy unoccupied molecular orbital (LUMO), and (**c**) highest energy occupied molecular orbital (HOMO).

The optimized structure of the coupled Na_2_Pc-TCNQ is presented in Figure 2a. This arrangement was directly obtained by means of geometry optimization of the species, in which both parts of the pair are located with a separation of 3 Å between the possible participants of the hydrogen bond. A weak interaction between phthalocyanine and TCNQ exists in the structure, which occurs through hydrogen bonds present at lengths of 2.94 and 3.13 Å. This is a similar interaction to that previously reported by Yoshida and coworkers [32]. The bonds are formed between the nitrogen atoms of TCNQ and the hydrogens of the periphery of the macrocycle, with the average energy for these bonds estimated from the Grimme’s correction at a value of 8.8 kcal/mol for each one. This strong value confirms the hydrogen bond interaction nature, considering that the Van der Waals values are smaller [33]. Additionally, the HOMO and LUMO calculated orbitals can be observed in Figure 2c,b respectively; the HOMO is practically over the Na_2_Pc, while the LUMO is completely on the TCNQ.

**Figure 3:**
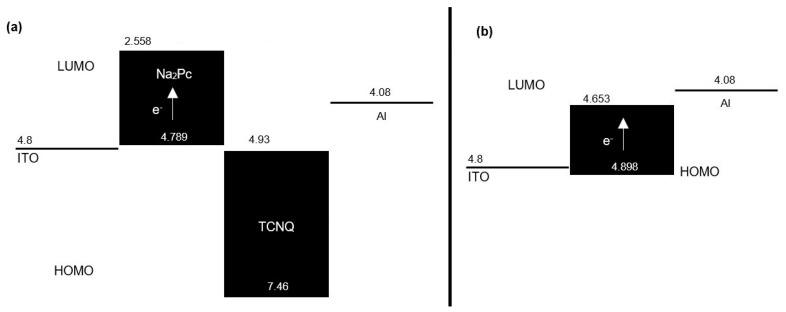
Energetic diagram for the devices of (**a**) flat heterojunction and (**b**) dispersed heterojunction.

In Table 1, the values calculated by DFT are shown for the highest energy occupied molecular orbital (HOMO) and the lowest energy unoccupied molecular orbital (LUMO) of phthalocyanine, TCNQ, and Na_2_Pc+TCNQ. Since in the devices both positive and negative charges are injected from the electrodes, it is important that the materials through which such charges must be transported have an optimal alignment of energy levels (Figure 3). For the flat heterojunction device (Figure 1a), it is desirable that the Na_2_Pc film, which contributes the holes for the charge transport, has a HOMO with a similar energy value to the work function of the anode, which it is in contact with it. As seen in the energy scheme of Figure 3a, the HOMO of Na_2_Pc with 4.789 eV is isoenergetic with the ITO work function of 4.8 eV. Conversely, the TCNQ film through which the electrons move and which is in contact with the cathode has a LUMO of 4.93 eV, while the work function of aluminum is 4.08 eV. This energetic difference (0.85 eV) transfers the charge between aluminum and the films that constitute the device. For the dispersed heterojunction device architecture (Figure 1b), this situation is different, as observed in the Figure 3b energetic scheme; although the work function of the anode (4.8 eV) is practically isoenergetic with the HOMO of the system Na_2_Pc+TCNQ (4.898 eV), the energetic gap between the LUMO (4.653 eV) and the work function of the cathode (4.08 eV) is 0.573 eV. This value is lower than the energetic gap between Al and the LUMO of TCNQ in the flat heterojunction device. Previous results indicated that charge transport is more efficient in the diverse heterojunction device and through the HOMO of the Na_2_Pc toward the LUMO of the TCNQ, however, electrical characterization in both devices must be carried out.

### 4.2. Characterization Study

**Figure 4:**
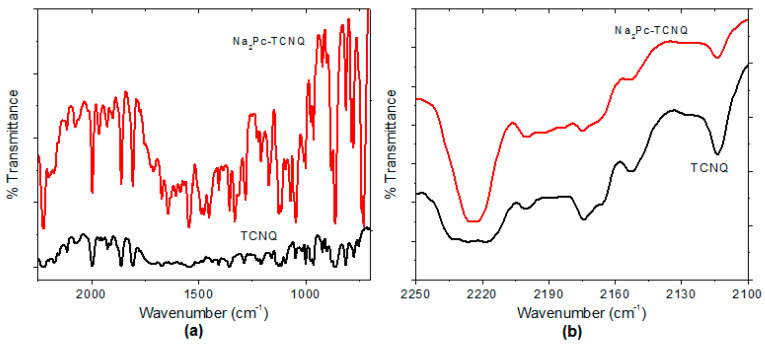
IR spectrum of Na_2_Pc+TCNQ and TCNQ in (**a**) the 700–2250 cm^−1^ region and (**b**) the cyano-stretching region.

IR spectroscopy was used to verify the main functional groups of Na_2_Pc+TCNQ and to prove that the films did not undergo degradation during deposition. Figure 4a shows the IR spectra for Na_2_Pc+TCNQ with respect to the Na_2_Pc. The band responsible for pyrrole in-plane stretch vibration in the phthalocyanine ring is observed at 1328 cm^−1^ and the bands located at 1278, 1170, and 1123 cm^−1^ result from the interaction of carbon with hydrogen atoms [34]. Also, the bands observed around 1604 and 1487 cm^–1^ result from a C=C stretching mode [34,35,36]. In addition, C≡N stretching bands of TCNQ appear around 2223 and 1670 cm^−1^ and the peaks around 1603, 1449, and 1207 cm^−1^ are related to C=C-H bending, C-CN stretching, and C=C ring stretching, respectively [37]. On the other hand, IR spectroscopy confirms the presence of the expected hydrogen bonds for Na_2_Pc-TCNQ. This bond is indirectly viewed by the strong absorbance at 2223 cm^−1^, which is characteristic of the lower frequency absorbance of conjugated nitriles [38,39]. Figure 4b shows the IR spectra in the CN stretching frequency region for Na_2_Pc+TCNQ in comparison with pure TCNQ. The Na_2_Pc+TCNQ spectrum reveals a CN stretching frequency at 2223 cm^−1^, while the TCNQ spectrum shows a broad signal between 2233 and 2217 cm^−1^. The change in the signal shape and amplitude could be related to the interaction between phthalocyanine and the TCNQ acceptor through hydrogen bonds [37,38,39].

**Figure 5:**
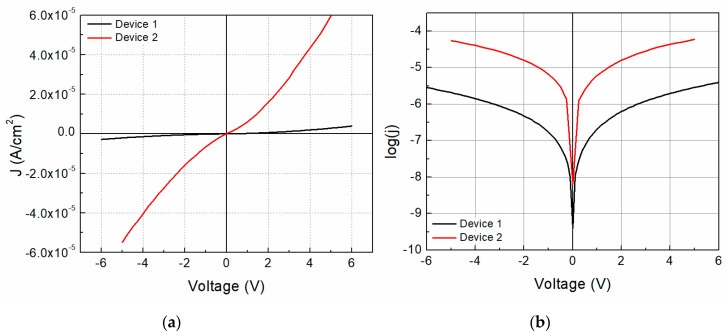
(**a**) J-V characteristics and (**b**) log(J)-V curves for the fabricated devices.

Figure 1 shows the different growth diode structures used for this work. The structure shown on Figure 1a corresponds to a planar heterojunction, whereas the structure shown in Figure 1b corresponds to a dispersed heterojunction. In the former, device 1 is constituted by a two-layered structure consisting of the TCNQ and the Na_2_Pc. On the other hand, device 2 is formed by a Na_2_Pc+TCNQ mixed single layer. For electrical characterization, the device was measured in a sandwich configuration. The selected voltage range was intended to not damage the device during measurement. Figure 5a shows the J-V characteristics of both devices. It is possible to observe a remarkable behavior change with the structure of this device; a diode type curve is observed for both devices and the curves in quadrants 1 and 3 are apparently symmetrical. Moderately larger current density values were achieved for the forward bias than in the reverse bias. The current density ranges for device 1 are from −2.83 × 10^−6^ to 3.92 × 10^−6^ A/cm^2^, whereas they exist from −5.49 × 10^−5^ to 5.96 × 10^−5^ A/cm^2^ for device 2 (−5 to 5 V interval), almost 28% and 8% variation, respectively. The latter indicates that the current density values of device 2 are considerably larger than those of device 1 and may be related to a charge carrier concentration augment by the total interface area variation, thereby affecting the recombination rate. Figure 5b shows the log(J)-V curve for both devices, where variation in the curves is observed. First, a possible increase in current at 0 volts is observed for device 2 with respect to device 1 from −9.40 to −8.18, almost 1 order of magnitude in current. Also, a change of ~1.5 orders of magnitude with the device construction is observed for higher voltages.

The current density for the space charge limited current (SCLC) region can be expressed as follows, since the electrodes form an ohmic contact with the organic semiconductor film:

(1) $$J_{\mathrm{SCLC}}=\frac{9\varepsilon_{r}\varepsilon_{0}{\mu V}^{2}}{8L^{3}}$$

where J is the current density, V is the applied voltage, μ is the mobility, L is the film thickness, and $\varepsilon_{r}$ and $\varepsilon_{0}$ are the relative material (3.6 for the CuPc [40]) and vacuum (8.85 × 10^−14^ Fcm^−1^) permittivity, respectively. The carrier mobility was calculated using Eq. 1 and fitting the slope of the J-V^2^ curve. The latter is because charge carriers injected from the contacts dominate the current on the J-V characteristics in the SCLC region, causing it to become quadratic; therefore, the current is only carrier mobility dependent. Table 2 contains the estimated mobility values for the devices in the SCLC region. The values show a large variation depending on the device construction, larger for device 2. The obtained mobilities values (10^−10^–10^−9^ m^2^/Vs) are of similar orders of magnitude as the ZnPc (10^−10^ m^2^/Vs), compared to previous reported results [16]. The change in the mobility is a consequence of the relationship of the TCNQ with the Na_2_Pc for the mixed film. The J-V characteristic also gives diode electrical property information, like the reverse saturation current and the ideality factor. Diode parameters were calculated for both devices and are included in Table 2. Among the parameters, the threshold voltage, shunt resistance (Rsh), saturation current (Is), saturation voltage (Vs), series resistance (Rseries) at 0.5 V, and Barrier Height (φ_b_) are observed. The forward current through the shottky contacts was determined using the following expression:

(2) $$I=I_{s}\exp\left(\frac{\mathrm{qV}}{\mathrm{nkT}}\right)$$

where V is the applied voltage, I_s_ is the saturation current, and n is the diode ideality factor. The saturation current was determined in the reverse bias using the following expression:

(3) $$I_{s}={\mathrm{AA}}^{*}\exp\left(\frac{-{q\varphi }_{b}}{\mathrm{kT}}\right)$$

where A is the diode field, A* is the effective Richardson constant (1.3 × 10^5^ A/cm^2^K^2^ for the ZnPc [41]), T is the absolute temperature, q is the electron charge, φ_b_ is the barrier height, and k is the Boltzman constant (8.6173 × 10^−5^ eV/K). The ideality factor can be calculated by the ln(J)-V linear region slope. Moreover, the barrier height can be calculated from the intersection of the line with the current axis and derived from the following expression:

(4) $$\varphi_{b}=\frac{\mathrm{kT}}{q}\left(\frac{{\mathrm{AA}}^{*}T^{2}}{I_{s}}\right).$$

The diode threshold voltage, defined as the voltage to start the driving current, is lower for device 2 (0.734 V), almost half the voltage for device 1 (1.203 V) when comparing both devices. This can be related to a reduction of the series resistance and interfacial trap levels. Also, it may indicate a reduction of the hole injection barrier on the ITO/TCNQ+Na_2_Pc interface. The observed value for device 2 is close to silicon diode voltage, whereas, for the shunt and series resistance, device 2 shows smaller values of ~10^4^ and 10^3^, respectively, compared to those of device 1 (~10^6^). The possible reduction of the series resistance for device 2 is a consequence of the homogenous mixture of the Na_2_Pc and TCNQ, which reduces the bulk resistance compared to the device 1 bilayer. The reduction of the number of interfaces located on device 1 (ITO/Na_2_Pc/TCNQ/Al) compared to device 2 (ITO/Na_2_Pc+TCNQ/Al) impact the series resistance reduction, dismissing the quality of the interfaces that also may affect the series resistance. Nevertheless, a reduction in shunt resistance, which governs the diode operation, may be triggered by parasitic currents related to crystalline structure defects (or deformation and stress) and impurities obtained during the deposition process. In the case of the saturation current and voltage, device 2 shows larger values of ~10^−5^ A and 0.78 V, respectively, compared to those of device 1, which shows values of ~10^−7^ A and 0.16 V, respectively, reflecting a change of approximately 2 orders of magnitude for the current and 5 orders of magnitude for the voltage. Moreover, a reduction in barrier height is observed for the mixed phase device, from 1.024 to 0.909 eV for device 1 and device 2, respectively. The observed barrier height is comparable to reported values for similar materials, like the NiPc with 0.96 eV [42]. The reduction in barrier height might be one of the reasons for the high current density observed through the device with a dispersed heterojunction Na_2_Pc+TCNQ film.

**Figure 6:**
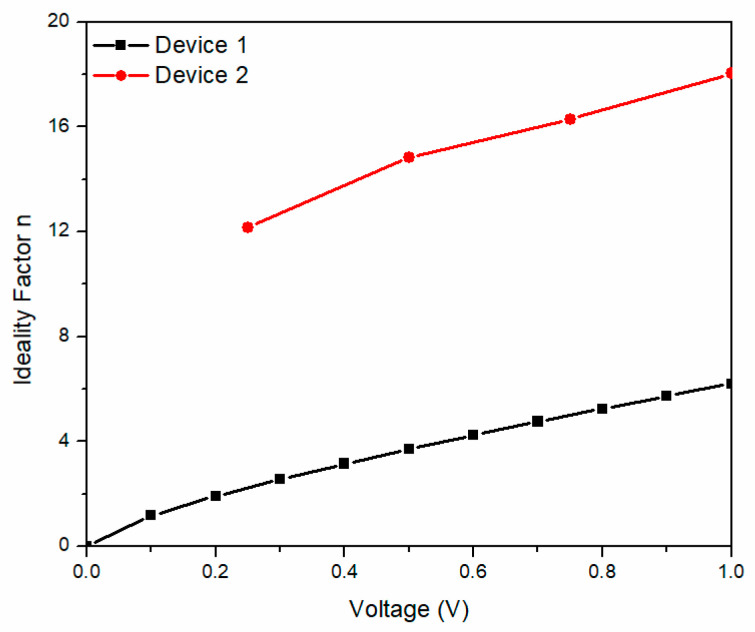
Voltage-dependent ideality factor of the devices.

The voltage-dependent ideality factor (n) of devices 1 and 2 is shown in Figure 6 for voltages ranging between 0 and 1 V. The n values are found to be relatively higher than the ideal diode (n = 1), where device 1 values are found between 0 and 6 and between 12 and 18 for device 2. A large difference in n is observed for both devices, the latter indicating that the diodes present a nonideal behavior as a possible consequence of the interfaces, no film homogeneity, and the observed series resistance. Also, changes in the ideality factor are consequences of different recombination mechanisms or a recombination rate change, indicating that device 2 suffers from high electron hole recombination in the depletion region, leading to a series resistance decrease. The change in the slope for both devices is because the shunt resistance dominates for lower voltages and the series resistance dominates for higher voltages, which is device construction sensitive. Cheung and Cheung defined the following expression [43]:

(5) $$\;\;H\left(I\right)=n\varphi_{b}+R_{s}I.$$

**Figure 7:**
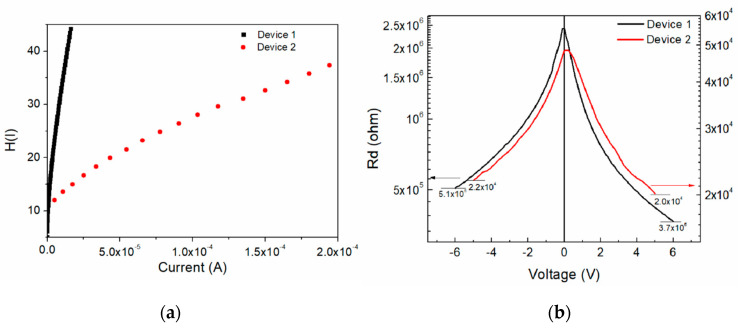
(**a**) Current-dependent H(I) and (**b**) voltage-dependent diode resistance of the devices.

Figure 7a shows the current-dependent H(I) for both devices. The H(I) slope is very different for the devices, where device 1 curve is steeper in slope than device 2. It is important to notice that an H(I) value of 35 was reached with a current of 2 × 10^−4^ A for device 2 and of 1.2 × 10^−5^ A for device 1. Figure 7b shows the voltage-dependent resistance across the diodes curve. The observed resistance is not symmetrical, and the curve varies depending on the device construction with respect to the applied voltage. For the reverse operation the curves demonstrate similar behavior, but for the forward operation the curve of device 1 shows variation while it decreases.

**Figure 8:**
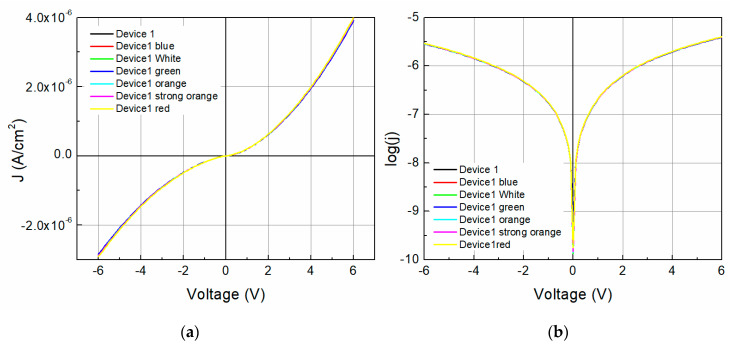
Device 1 (**a**) J-V characteristics and (**b**) log(J)-V curves under illumination.

**Figure 9:**
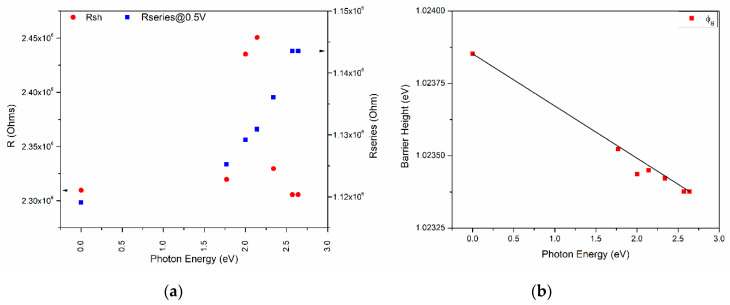
(**a**) Series and shunt resistance, threshold voltage, and (**b**) barrier height against illumination photon energy for device 1.

To address the effect of the incident light on device 1 and its photosensitivity, the devices’ J-V characteristics and log(J)-V curves under illumination (six light colors) were obtained and are shown in Figure 8. The reverse current presents a slightly higher effect with the wavelength than the forward operation due to a difference in electronic band transition. Also, the current density at 0 V, which is mostly related to the short circuit current (Isc), changes with the incident light with no specific trend, even up to 9.39 (darkness) and 9.88 (blue light). The series resistance and shunt resistance were calculated and plotted along with the different incident lights for device 1, as shown in Figure 9. Different behaviors for both resistances exist as the incident photon energy increases. The device shunt resistance increases for values higher than 1.75 eV and decreases again (>2.25 eV) to similar resistance values as those of the darkness state, whereas the values increase with the incident photon energy for the series resistance. For instance, a variation of ~160 × 10^3^ Ω for the shunt resistance and ~20 × 10^3^ Ω for the series resistance can be observed. Figure 9b shows the effect on the barrier height caused by the incident light photon energy. The barrier height decreases with the photon energy from 1.024 eV to 1.023 eV with a fitted slope of −1.804 × 10^−4^, with the highest variation as much as almost 0.001 eV.

**Figure 10:**
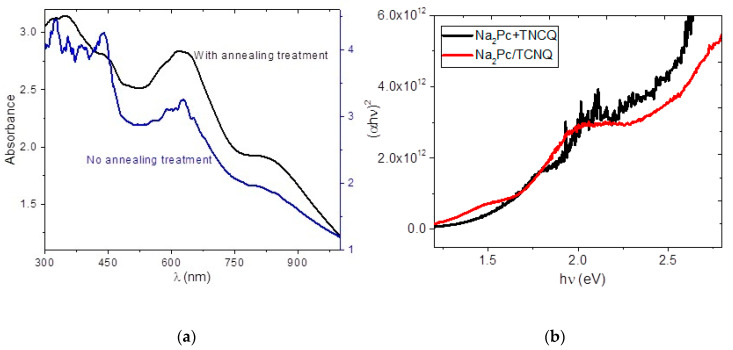
(**a**) UV-vis spectrum of Na_2_Pc/TCNQ and (**b**) Tauc plot of Na_2_Pc/TCNQ and Na_2_Pc+TCNQ films.

Thermal annealing was conducted for device 1 to evaluate possible improvement of the device’s electrical properties. Table 2 shows the calculated results and comparison with an untreated device. The threshold voltage is reduced from 1.203 V to 1.147 V. The shunt resistance and series resistance show a small variation from 2.31 × 10^6^ Ω to 2.30 × 10^6^ Ω and from 1.12 × 10^6^ Ω to 1.14 × 10^6^ Ω, respectively. The latter is related to the slight decrease in observed barrier height. All of these results may be due to long-range molecular stacking characteristic of the crystallization of organic thin films, with an excess of electron current flow involved compared to the hole, resulting in a poor recombination rate. In order to complement the above and to obtain the optical properties, the UV-vis spectroscopy of the system Na_2_Pc/TCNQ was carried out before and after annealing; the results are shown in the spectra of Figure 10a. After sequential deposit, the presence of the two films prevent adequate measurement for the sample without annealing. However, in the spectrum the two typical bands of phthalocyanine, a Q band and a Soret band are observed. The peak around 628 nm corresponds to the *Q*-band transition of the Na_2_Pc, assigned to the degenerate transition $a_{1u}\left(\pi \right)\to e_{g}\left(\pi^{*}\right)$ [44,45]. The wavelength of the Soret band is not possible to exactly determine but lies within a wide range between 327 and 430 nm. On the other hand, after receiving the annealing treatment, a noticeable improvement in the UV-vis spectrum is observed. The Q band appears with the signal at 616 nm [44,45], assigned to the first π-π* transition on the Na_2_Pc [35,36]. The Soret band is observed at 343 nm [35,44,45] and is due to $a_{2u}\left(\pi \right)\to e_{g}\left(\pi^{*}\right)$ together with $b_{2u}\left(\pi \right)\to e_{g}\left(\pi^{*}\right)$ transitions in Na_2_Pc [45,46,47]. It is important to consider that the films *Q*-band is sensitive to both the Na_2_Pc molecular structure and the structure of their films [24,36,48]. The *Q*-band exhibits a red shift, which is typically observed for slipped, coafacially stacked, macrocycle configurations [24]. Additionally, the UV-vis spectrum splits into two bands with the maxima at 616 nm and 802 nm, corresponding to the triclinic and monoclinic phases mixture in Na_2_Pc [24,49]. Based on these results, direct transitions related to polycrystalline films are assumed to be dominant in the Na_2_Pc/TCNQ system, which was verified by the calculation of the optic band gap (*E_opt_*). The *E_opt_* is determined through extrapolation of the straight-line graphs to zero absorption observed in the spectral dependence of (*αhν*)^2^ over a range of photon energies (*hν*) (see Figure 10b) [50]. The absorption coefficient (*α*) and the frequency (*ν*) are experimentally obtained from the UV-vis spectrum of the film, while *h* is Planck’s constant. The coefficient *α* near the band edge shows an exponential dependence upon photon energy, which usually obeys the Urbach relationship $\alpha h\nu =\beta {\left(h\nu -E_{g\;}\right)}^{n}$, and the value of *n* characterizes the optical absorption process, which theoretically equals ½ and 2, depending on the transitions, e.g., direct allowed and indirect allowed transitions, respectively [50]. In the present study, the linear dependence of (α*hν*)^2^ on *hν* is plotted in Figure 10b. The plot indicates two regions, one with higher energy, called the fundamental gap, at 2.01 eV and the other with a lower energy gap, called the onset gap, at 1.43 eV [35,36,42]. The experimental gap values were obtained in a similar way for the Na_2_Pc+TCNQ system, with results of 1.6 and 2.5 eV for the onset gap and the fundamental gap, respectively. When comparing the curves in Figure 10b, minimal differences are observed, indicating the flat heterojunction structure may present behavior similar to that of the system with dispersed heterojunction after being treated. However, when comparing the experimental gap to the theoretical gap (E_theo_) of 0.245 eV, the latter is the lowest value (indeed, this value corresponds to a conductor species and not to a semiconductor), since it does not consider external effects of the semiconductor molecules, such as the presence of impurities and degree of stacking of the electron donor and acceptor molecules. The dispersed heterojunction semiconductor device based on Na_2_Pc and TCNQ offers promising low-cost, lightweight, and flexible diode characteristics. The Na_2_Pc donor and the TCNQ acceptor form phases which provide channels to carry photogenerated charge carriers. This heterojunction system exhibits a donor–acceptor phase separation on a very small, bicontinuous scale. Each interface may be within a distance less than the exciton diffusion length from the absorbing site, thereby promoting better charge transport within the device.

## 5. Conclusions

Flat heterojunction (device 1) and dispersed heterojunction (device 2) flexible devices were studied with Na_2_Pc and tetracyano-π-quinodimethane films. The Na_2_Pc is rarely used but shows interesting optoelectronic properties for electronic device applications. Theoretical calculations based on DFT with dispersion force analysis show that the electronic relationship between phthalocyanine and TCNQ is through hydrogen bonds with bond lengths of 2.94 y 3.13 Å. Device 2 current density values are considerably larger than those of the device 1. The electrical properties are improved in the dispersed heterojunction device. A forward bias current density exists of up to 3.92 × 10^−6^ A/cm^2^ and 5.96 × 10^−5^ A/cm^2^ for device 1 and 2 respectively, almost 28% and 8% variation, respectively. A current change from −9.40 to −8.18 A, of almost 1 order of magnitude is observed at 0 V, with the obtained mobilities being 10^−10^–10^−9^ m^2^/Vs. The change in the mobility is a consequence of the relationship of the TCNQ with the Na_2_Pc for the mixed film. A reduction in barrier height was observed for the mixed phase device, from 1.024 to 0.909 eV. The reduction in barrier height might be related to the higher current density through the device with the dispersed heterojunction TCNQ+Na_2_Pc film. The ideality factor values are relatively higher than the ideal diode (n = 1), a consequence of the interface and no film homogeneity. The series resistance values are increased with the incident photon energy, while the shunt resistance does not show a specific behavior. By means of thermal annealing of device 1, a small electrical property effect is observed, with the threshold voltage decreasing from 1.203 V to 1.147 V, φ_b_ decreasing by 0.001 eV, and the shunt and series resistance showing a small variation. After heat treatment, the flat heterojunction system shows a slightly lower optical gap (1.43 eV) than that presented by the disperse heterojunction system (1.6 eV). However, this device does not need to be annealed, so it offers a more efficient and lower cost option for flexible electronics.

## Figures and Tables

**Table 1:** HOMO, LUMO, and theoretical band gap.

Sample	HOMO (eV)	LUMO (eV)	Band Gap (eV)
Na_2_Pc	−4.789	−2.558	2.230
TCNQ	−7.460	−4.930	2.500
Na_2_Pc+TCNQ	−4.898	−4.653	0.245

**Table 2:** Electronic parameters in devices.

Item	Device 1 Darkness	Device 1 Annealed Darkness	Device 2 Darkness
Threshold voltage [V]	1.203	1.147	0.734
Rsh [Ω]	2.31 × 10^6^	2.30 × 10^6^	4.82 × 10^4^
Is [A]	3.06 × 10^−7^	3.11 × 10^−7^	2.51 × 10^−5^
Vs [V]	0.158	0.144	0.784
Rseries (@0.5V) [Ω]	1.12 × 10^6^	1.14 × 10^6^	8.61 × 10^3^
Barrier height (φ_b_) [ eV]	1.024	1.023	0.909
Mobility [m^2^/Vs]	1.38 × 10^−10^	1.40 × 10^−10^	2.92 × 10^−9^
